# Risk factors of progressive IgA nephropathy which progress to end stage renal disease within ten years: a case–control study

**DOI:** 10.1186/s12882-016-0429-x

**Published:** 2017-01-07

**Authors:** Danhua Shu, Feifei Xu, Zhen Su, Ji Zhang, Chaosheng Chen, Jianna Zhang, Xiaokai Ding, Yinqiu Lv, Haixia Lin, Peipei Huang

**Affiliations:** Department of Nephrology, the First Affiliated Hospital of Wenzhou Medical University, Wenzhou, China

**Keywords:** IgA nephropathy, Oxford classification, End stage renal disease, Follow-up clinical data

## Abstract

**Background:**

There were few related studies aiming to severe IgA nephropathy (IgAN) which could progress rapidly to end stage renal disease (ESRD) within ten years. To find valuable clinical or pathological factors and promising precautions is essential.

**Methods:**

A single center case–control study was performed. Fifty ESRD patients with the primary cause of IgAN and a short renal survival time of less than ten years after diagnose were enrolled in the case group. One hundred IgAN patients with a renal survival time of more than ten years were enrolled in the control group. IgA Oxford classification scores, clinical data at baseline and during the follow-up were collected. Multivariate logistic regression was used to investigate factors associated with the development of ESRD.

**Results:**

There were significant differences in baseline clinical data between these two groups, as well as the constituent ratio of Oxford MEST-score. Distinct differences were observed in time-average uric acid(TA-UA), time-average hemoglobin(TA-Hb), time-average albumin(TA-Alb), time-average total cholesterol(TA-TC) and time-average urinary protein(TA-P) during the follow-up. In multivariate logistic models, IgA Oxford score M1(OR = 5.10, *P* = 0.018) and eGFR(OR = 0.97, *P* = 0.039) at biopsy, TA-UA (OR = 2.06, *P* = 0.026) and TA-Hb (OR = 0.53, *P* = 0.022) during the follow-up were identified independent factors for developing ESRD.

**Conclusion:**

IgAN patients with pathological assessment of M1, low baseline eGFR, TA-Hb and high TA-UA were more likely to progress to ESRD, and should be paid more attention. Appropriate regulations of UA, Hb and urine protein after diagnose may be a promising treatment.

**Electronic supplementary material:**

The online version of this article (doi:10.1186/s12882-016-0429-x) contains supplementary material, which is available to authorized users.

## Background

IgAN is one of the most common glomerulonephritis in Asian, accounting for 45.26% of primary glomerular diseases in China, and is also a leading cause of ESRD. Most of the IgAN patients showed good outcomes, but about 30% of them progressed to ESRD within 10–20 years, while in some patients the disease developed rapidly to ESRD within ten years. Clinical and pathological markers, such as proteinuria, hypertension, decreased eGFR at the time of biopsy and the MEST score were found to be associated with renal outcome in previous studies [1, 2]. It was widely accepted that clinical and pathological markers above were significant factors for IgAN development. Compared with numerous studies on MEST score and clinical data at biopsy, follow-up features, except for urine protein, were paid little attention. Even then those follow-up data were still of great importance [3, 4]. The main purpose of this study was to assess the prognostic value of these follow-up clinical data, including TA-P, TA-UA, TA-Alb, TA-Hb and TA-TC, on the progression of progressive IgAN and also to reassess the predictive value of MEST score and clinical features at biopsy. This could be practical to the full stage guidance of IgAN treatment.

## Methods

### Study design and participants

This was an observational case–control study. Fifty end stage renal disease patients with the primary cause of IgAN and a short renal survival time of less than ten years after renal biopsy were enrolled in case group. One hundred IgAN patients with a renal survival time of more than ten years after biopsy were reviewed as control group. Patients enrolled in this study were given the diagnosis of primary IgAN between 1997 and 2012 in the First Affiliated Hospital of Wenzhou Medical University. Exclusion criteria for both groups included: renal biopsy conducted in other hospitals, a secondary cause of IgAN, such as Henoch-Schonlein purpura, systemic lupus erythematosus, chronic liver disease and other autoimmune disorders, aged <18 years at biopsy and history of cardiovascular events, carotid artery surgery or any organ transplantation.

### Clinical and pathological data

Baseline clinical data, including gender, age, sCr, Hb, Alb, UA, TC, 24 h urine protein, hypertension and self-reported hematuria history, were obtained from the original medical records of each patient. Follow-up clinical data, including sCr, UA, Alb, Hb, TC and urine protein levels as well as the observation date at each checkup before the point of ESRD in cases or the last follow-up in controls were recorded. Renal survival time was accounted from biopsy to the point of ESRD or the last follow-up. For each patient, the average level (time-weighted average) of each clinic feature (UA, Alb, Hb, TC and urinary protein) were calculated from the area under the curve of serial measurements standardized by the length of the study. The kidney specimen of every patient was reviewed according to the Oxford classification [5]. eGFR was calculated by modified MDRD equation: eGFR[ml · min^−1^ · (1.73 m^2^)^−1^] = 175 × [Scr, mg/dl]^-1.234^ × [age]^-0.179^[×0.79(if female)]. ESRD was defined as eGFR < 15 ml · min^−1^ · (1.73 m^2^)^−1^ or initiation of dialysis or transplantation. During the follow-up, most of the urinary protein tests were semi-quantified with a standard urine dipstick with (−), (±), (+), (++), (+++) and (++++) corresponding to <0.1, 0.1-0.2, 0.2-1.0, 1.0-2.0, 2.0-4.0 and >4.0 g/L of urine albumin, respectively. In this study we quantified (−) (±) to 0, (+) to 0.6, (++) to 1.5, (+++) to 3, (++++) to 4 g/L [6], respectively.

### Statistical analysis

Statistical analysis was performed using SPSS17.0 software. Qualitative variables were expressed as number and percentage, compared using the unpaired t-test (Student’s t-test), the Mann–Whitney U test or Spearman correlation. Continuous variables were expressed as mean values ± standard deviation when normally distributed, or median(range) when not, and data were compared using the chi-square test or Fisher’s exact test or the Mann–Whitney U test, one-way ANOVA or Pearson correlation. Univariate logistic regression and multivariate logistic regression were used to determine factors to outcomes. A significance level of 0.05 was accepted.

## Result

### Baseline clinical data

A total of one hundred and fifty patients with a primary cause of IgAN were enrolled in this study. The median renal survival time in case group (fifty cases) was 67 months (1–120 months) with the initial CKD stage at the time of renal biopsy: CKD I 8 cases (16.0%), CKD II 12 cases (24.0%), CKD III 23 cases (46.0%), CKD IV 7 cases (14.0%). In control group (one hundred cases), the median renal survival time was 132 months (112–200 months), with the initial CKD stage at biopsy: CKD I 55 cases (55%), CKD II 35 cases (35%), CKD III 9 cases (9%), CKD IV 1 case (1%).

The case group had significantly higher frequencies of M1, S1, T1 and T2 score (*P* < 0.05) and higher levels of sCr, UA, TC, 24 h urinary protein and the proportion of hypertension at biopsy than in control group (*P* <0.05), while Hb, Alb, eGFR and the proportion of hematuria history were lower than those in control group (*P* <0.05) (Table 1).

**Table 1:** Baseline clinical data

Clinical data	Case group *n* = 50	Control group *n* = 100	Oxford classification	Case group Cases (%)	Control group Cases (%)
At time of biopsy			Mesangial hypercellularity **		
Gender (M/F)	35/15	56/44	M0	11 (22.0%)	72 (72.0%)
Age (y)	35.8 ± 9.9	35.4 ± 10.1	M1	39 (78.0%)	28 (28.0%)
eGFR	57.3 ± 27.6**	95.7 ± 32.7	Endocapillary hypercellularity		
sCr (mg/dl)	1.4 (0.8–4.3)**	0.9 (0.5–2.1)	E0	14 (28.0%)	32 (32.0%)
UA (mg/dl)	8.0 (1.9–15.3)**	5.7 (2.3–12.9)	E1	36 (72.0%)	68 (68.0%)
Hb (g/dl)	12.2 ± 2.0*	12.9 ± 1.8	Segmental Glomerulosclerosis*		
Alb (g/dl)	3.5 ± 0.8*	3.9 ± 0.6	S0	12 (24.0%)	52 (52.0%)
TC (mg/dl)	207.7 (111.5–392.3)*	184.6 (100.0–369.2)	S1	38 (76.0%)	48 (48.0%)
Proteinuria (g/day)	2.9 (0.1–8.3)**	0.8 (0–9.9)	Tubular atrophy/Interstitial fibrosis**		
Hypertension	31 (62.0%)*	47 (47.0%)	T0	16 (32.0%)	86 (86.0%)
Macro-hematuria	2 (4.0%)*	18 (18.0%)	T1	26 (52.0%)	11 (11.0%)
			T2	8 (16.0%)	3 (3.0%)

*eGFR* estimated glomerular filtration rate (ml · min-1 · (1.73 m2)-1), *sCr* serum creatinine, *UA* uric acid, *Hb* hemoglobin, *Alb* serum albumin, *TC* total cholesterol

*P < 0.05; **P < 0.001

### Clinical features during the follow-up

**Fig. 1:**
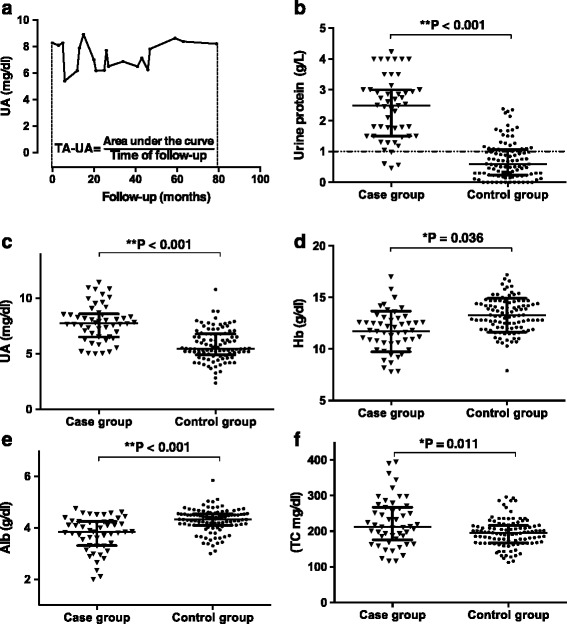
Clinical features during follow-up. **a**, Serial measurements of proteinuria during follow-up in one patient and the definition of TA-P for this patient. Comparison of TA-P (**b**), TA-UA (**c**), TA-Hb (**d**), TA-Alb (**e**) and TA-TC (**f**). Dotted lines present the normal range of each clinical feature. Median and interquartile range were presented in each figure

Average levels of each clinical feature during the follow-up were calculated and compared. Figure 1a shows an example of serial measurements of UA during the follow-up of one patient and the equation to calculate the TA-UA in current study [7]. Similarly, TA-P, TA-Hb, TA-Alb and TA-TC of each patient were calculated by the same method. As shown in Fig. 1, there were remarkable differences in TA-P [2.5 (0.5–4.2) versus 0.6 (0–2.4) g/L; *P* <0.001], TA-UA [7.8 ± 1.7 versus 5.8 ± 1.4 mg/dl; *P* <0.001], TA-Hb [11.7 ± 2.0 versus 13.3 ± 2.0 g/dl; *P* <0.001], TA-Alb [3.8 (2.0–4.7) versus 4.3 (3.0–5.8) g/dl; *P* <0.001] and TA-TC [212.3 (116.0–394.4) versus 194.7 (112.1–296.2) mg/dl; *P* = 0.011] between cases and controls.

### Risk assessment by univariate and multivariate logistic analysis

Clinical and pathological data were analyzed by logistic regression to identify whether were they associated with developing of ESRD among those patients (Table 2), whereas TA-P was excluded here because of its inaccurate property. TA-P in this study was estimated from semi-quantified urine protein measurement, which may not be able to reflect the accurate level of urine protein compared with albumin creatinine ratio or 24 h urine protein measurement, and may cause statistical bias in multivariate analysis. Univariate logistic analysis indicated that Oxford classification: M1, S1, T1 or T2, high UA, TC, 24 h urine protein levels, the hypertension history at biopsy and high TA-UA, TA-TC during the follow-up were associated with an increased risk for developing ESRD, while high levels of baseline eGFR, Hb and Alb, TA-Hb and TA-Alb during the follow-up and the present of macro-hematuria were associated with a decreased risk of ESRD. The risk to develop ESRD of all these clinical and pathological features were further assessed in multivariate logistic regression analysis. The result showed: M1(OR = 5.10, *P* = 0.018), eGFR at biopsy (OR = 0.97, *P* = 0.039), TA-UA (OR = 2.06, *P* = 0.026) and TA-Hb (OR = 0.53, *P* = 0.029) were independent factors for developing ESRD among IgAN patients.

**Table 2:** Predictive factors for ESRD by univariate and multivariate logistic regression

	Univariate Logistic		Multivariate Logistic	
	OR (95%CI)	P	OR (95%CI)	P
Mesangial hypercellularity				
M0	1		1	
M1	9.12 (4.10–20.27)	<0.001	5.10 (1.33–19.60)	0.018
Endocapillary hypercellularity				
E0	1		1	
E1	1.21 (0.57–2.55)	0.617	1.62 (0.33–8.02)	0.557
Segmental Glomerulosclerosis				
S0	1		1	
S1	3.43 (1.61–7.32)	0.001	0.57 (0.12–2.74)	0.480
Tubular atrophy/interstitial fibrosis				
T0	1		1	
T1	12.71 (5.25–30.76)	<0.001	1.82 (0.33–10.0)	0.492
T2	14.33 (3.43–59.91)	<0.001	0.32 (0.02–5.48)	0.431
At the time of biopsy				
eGFR	0.96 (0.94–0.97)	<0.001	0.97 (0.93–0.998)	0.039
Gender (male)	1.83 (0.89–3.78)	0.100	5.38 (0.82–35.49)	0.081
Age	1.0 (0.97–1.04)	0.830	0.92 (0.83–1.01)	0.087
UA	1.74 (1.39–2.16)	<0.001	0.94 (0.59–1.50)	0.795
Hb	0.82 (0.68–0.99)	0.039	1.14 (0.60–2.17)	0.692
Alb	0.40 (0.24–0.67)	0.001	0.53 (0.18–1.62)	0.267
TC	1.01 (1.0–1.02)	0.006	1.003 (0.99–1.02)	0.687
24 h urine protein	1.60 (1.30–1.96)	<0.001	1.17 (0.86–1.58)	0.313
Hypertension (yes)	1.84 (0.92–3.68)	0.085	4.33 (0.79–23.80)	0.092
Macro-hematuria (yes)	0.19 (0.04–0.85)	0.030	0.05 (0–9.02)	0.264
Follow-up				
TA-UA	2.36 (1.74–3.19)	<0.001	2.06 (1.09–3.90)	0.026
TA-Hb	0.61 (0.48–0.76)	<0.001	0.53 (0.30–0.91)	0.029
TA-Alb	0.18 (0.08–0.38)	<0.001	0.48 (0.10–2.37)	0.367
TA-TC	1.01 (1.0–1.02)	0.002	1.004 (0.99–1.02)	0.697

*OR* odds ratio, *95% CI* 95% confidence interval

## Discussion

IgAN is the leading cause of adult primary glomerulonephritis progressing to ESRD [8]. However, there is still a lack of specific therapeutic interventions, because of the unclearness of its etiology and pathogenesis, so early identification of risk factors and timely treatment may be essential to the renal outcome. Progressive IgAN, which progress to ESRD within ten years, only accounted for a small part of IgAN population and normally were studied along with patients under stable condition, for instance, remaining normal renal function and slowly developing sCr, by retrospective cohort study [6, 9], which means some characteristics of progressive IgAN may be covered by the stable one. In this study, as the main object, progressive IgAN patients were compared with patients under stable renal conditions to identify the risk factors of poor prognosis, aiming to provide a promising guidance for the further treatment.

Impaired renal function, consistent proteinuria with more than 1 g per day and Oxford pathological score T1/T2 were recognized as independent risk factors for IgAN in a couple of previous studies [5, 7]. However, a great number of those founding was based on clinical and pathological data at the time of biopsy, with the absence of follow-up data. In this study, those factors were further considered with the present of follow-up features. In statistical practice (Additional file 1), variables were put into the logistic regression mode one by one to dig underlying information between them. When only MEST scores were included in the multivariate logistic analysis (Additional file 1, mode 1), M1, T1 and T2 were adverse prognostic factors to ESRD, but the OR value of them decreased and even showed no significance with the entrance of eGFR at biopsy (Additional file 1, mode 2). This could be explained by the strong correlation between MEST score and eGFR (Additional file 2), indicating eGFR may be a better predictor for renal outcome than T2 in severe IgAN. This situation maintained until the participant of demographic and baseline laboratorial data, including gender, age, UA, Hb, Alb, TC and 24 h urinary protein (Additional file 1, mode 8), in which score T and eGFR lost its position and gender (male) appeared to be a strong risk factor to unfavorable prognosis. The complex cross-relation among score T, gender, baseline eGFR, UA, Hb and Alb may contribute to this result (Additional file 2). In addition, there are innate difference between males and females in the normal range of UA and Hb. The situation changed again when follow-up clinical data were enrolled (Additional file 1, mode 11). Then eGFR came back to the predictor group instead of gender. This mode showed the relevance of follow-up clinical features more than those at biopsy. TA-P, though not that credible, showed a significant impact on renal outcome in mode 15 (Additional file 1). In general, stepwise logistic analysis illustrated that correlations between clinical data were inevitable and results of statistical analysis can be various with different variates in the analysis mode, and multivariate analysis was a proper way to distinct the strongest factors.

In previous studies, Oxford pathological score T was highly recognized as an important predictor of developing ESRD [9, 10], while roles M, E and S played were controversy. The Oxford score of 1026 primary IgAN patients from 18 clinical research centers in China were compared with the data in the original IgAN Oxford classification study [11]. Then a higher ratio of M1 and E1 and a lower ratio of S1 were observed in Chinese population, while the proportion of T1/T2 between them was similar. In this study, M1, T1 and T2 were identified as risk factors when only Oxford score were included in the logistic regression (Additional file 1, mode 1), but only M1 remained as an independent factor in multivariate analysis. Although T did not maintain a marked role in multivariate analysis, it was also of great importance because of its correlation with most variates in this study (Additional file 2). Combined with the results, we came up with a hypothesis that pathological score M1 might be a risk factor for progressing to ESRD in Chinese IgAN population.

There was an increasing attention on the relationship between hyperuricemia [12] and chronic kidney disease [13]. High concentration of serum UA may lead to nephropathy and renal function impairment by directly mechanical damage or indirectly inflammation and cell phenotype inversion, resulting in the reduction of UA excretion and forming a vicious cycle. Normal ranges of Hb and Alb were also reported beneficial to stable internal environment and normal physiological activities [14, 15]. Theories above could provide our result with support that serum UA and Hb levels during the follow-up can significantly affect the progress of IgAN to ESRD. Although TA-P was excluded from the final multivariate analysis for the accuracy of the mode, the importance of it cannot simply be ignored (Additional file 1, mode 15) and it had also long been recognized as a strong and reliable factor to renal outcome [16, 17].

Our research did not only come up with a similar result with others that M1, T1/T2 and eGFR at biopsy were strongly related to IgAN outcome, but also first assess the role TA-UA, TA-Hb, TA-Alb and TA-TC played in IgAN progressing and come up with a clinically significant result that TA-UA and TA-Hb were independent factors for developing ESRD. One of the limitation of this study was its retrospective nature, which means the data we collected was restricted. There was also a selection bias: patients enrolled were those who followed up in our hospital for a long term, so they might not be a fully presentative sample of the general population. A long-term prospective study or a clinical trial may be better for the study of IgAN treatment.

## Conclusions

In summary, patients with pathological assessment of M1, T1 or T2, an impaired renal function, abnormal blood biochemical parameters and hypertension at biopsy should be paid more attention, and therapies aiming to keep UA and Hb levels under the control and reduce urinary protein during the follow-up are highly recommended. Pathological type M may play an important role in IgAN outcomes among Chinese population.

## Additional files

Additional file 1: Stepwise multivariate logistic analysis. Contains detailed results in every step of stepwise multivariate logistic analysis. (DOCX 73 kb)
Additional file 2: Correlation between variates. Correlation analysis between each variate involved in this study. (DOCX 40 kb)

## Abbreviations

Alb: Serum albumin
eGFR: Estimated glomerular filtration
ESRD: End stage renal disease
Hb: Hemoglobin
IgAN: IgA nephropathy
sCr: Serum creatinine
TA-Alb: Time-average albumin
TA-Hb: Time-average hemoglobin
TA-P: Time-average urinary protein
TA-TC: Time-average total cholesterol
TA-UC: Time-average uric acid
TC: Total cholesterol
UA: Uric acid(UA)
