# Supplementary material for: Risk factors of progressive IgA nephropathy which progress to end stage renal disease within ten years: a case–control study
Source: BMC Nephrol. 2017 Jan 7;18:11. doi: 10.1186/s12882-016-0429-x (PMC5219698; doi:10.1186/s12882-016-0429-x)
Supplement: Additional file 1: — Stepwise multivariate logistic analysis. Contains detailed results in every step of stepwise multivariate logistic analysis. (DOCX 73 kb) [file 12882_2016_429_MOESM1_ESM.docx]

Additional file 1 Stepwise multivariate logistic analysis

|  | Mode 1 |  | Mode 2 |  | Mode 3 |  | Mode 4 |  | Mode 5 |  |
| --- | --- | --- | --- | --- | --- | --- | --- | --- | --- | --- |
|  | OR(95%CI) | P | OR(95%CI) | P | OR(95%CI) | P | OR(95%CI) | P | OR(95%CI) | P |
| Mesangial hypercellularity |  |  |  |  |  |  |  |  |  |  |
| M1 | 7.53 (2.94-19.30) | < 0.001 | 6.40 (2.40-17.07) | <0.001 | 6.13 (2.23-16.80) | <0.001 | 5.80 (2.09-16.11) | 0.001 | 5.52 (1.96-15.57) | 0.001 |
| Endocapillary hypercellularity |  |  |  |  |  |  |  |  |  |  |
| E1 | 1.37 (0.50-3.73) | 0.536 | 0.88 (0.29-2.61) | 0.811 | 0.995 (0.31-3.25) | 0.994 | 0.93 (0.28-3.11) | 0.902 | 0.76 (0.21-2.70) | 0.666 |
| Segmental Glomerulosclerosis |  |  |  |  |  |  |  |  |  |  |
| S1 | 1.28 (0.46-3.60) | 0.634 | 1.61 (0.55-4.73) | 0.385 | 1.70 (0.55-5.23) | 0.356 | 1.63 (0.52-5.09) | 0.404 | 1.87 (0.58-5.97) | 0.293 |
| Tubular atrophy/interstitial fibrosis |  |  |  |  |  |  |  |  |  |  |
| T1 | 10.50 (3.84-28.71) | < 0.001 | 4.44 (1.43-13.79) | 0.010 | 4.49 (1.36-14.86) | 0.014 | 4.10 (1.23-13.64) | 0.021 | 3.58 (1.02-12.59) | 0.046 |
| T2 | 10.06 (1.98-51.19) | 0.005 | 2.57 (0.39-16.80) | 0.325 | 2.33 (0.28-19.17) | 0.430 | 2.13 (0.25-18.23) | 0.491 | 1.57 (0.16-15.42) | 0.698 |
| At the time of biopsy |  |  |  |  |  |  |  |  |  |  |
| eGFR |  |  | 0.97 (0.95-0.99) | 0.005 | 0.97 (0.95-0.99) | 0.004 | 0.97 (0.95-0.99) | 0.014 | 0.97 (0.95-0.995) | 0.020 |
| Gender (male) |  |  |  |  | 2.39 (0.81-6.99) | 0.113 | 1.95 (0.62-6.13) | 0.253 | 3.24 (0.86-12.24) | 0.084 |
| Age |  |  |  |  | 0.97 (0.92-1.02) | 0.256 | 0.97 (0.92-1.02) | 0.250 | 0.97 (0.91-1.02) | 0.217 |
| UA |  |  |  |  |  |  | 1.16 (0.87-1.53) | 0.314 | 1.21 (0.90-1.61) | 0.209 |
| Hb |  |  |  |  |  |  |  |  | 0.77 (0.57-1.04) | 0.092 |
| Alb |  |  |  |  |  |  |  |  |  |  |
| TC |  |  |  |  |  |  |  |  |  |  |
| 24h urine protein |  |  |  |  |  |  |  |  |  |  |
| Hypertension (yes) |  |  |  |  |  |  |  |  |  |  |
| Macrohematuria (yes) |  |  |  |  |  |  |  |  |  |  |
| Follow-up |  |  |  |  |  |  |  |  |  |  |
| TA-UA |  |  |  |  |  |  |  |  |  |  |
| TA-Hb |  |  |  |  |  |  |  |  |  |  |
| TA-Alb |  |  |  |  |  |  |  |  |  |  |
| TA-TC |  |  |  |  |  |  |  |  |  |  |
| TA-P |  |  |  |  |  |  |  |  |  |  |

continued

|  | Mode 6 |  | Mode 7 |  | Mode 8 |  | Mode 9 |  | Mode 10 |  |
| --- | --- | --- | --- | --- | --- | --- | --- | --- | --- | --- |
|  | OR(95%CI) | P | OR(95%CI) | P | OR(95%CI) | P | OR(95%CI) | P | OR(95%CI) | P |
| Mesangial hypercellularity |  |  |  |  |  |  |  |  |  |  |
| M1 | 5.40 (1.88-15.48) | 0.002 | 5.62 (1.89-16.67) | 0.002 | 5.86 (1.90-18.10) | 0.002 | 5.83 (1.87-18.17) | 0.002 | 6.13 (1.95-19.32) | 0.002 |
| Endocapillary hypercellularity |  |  |  |  |  |  |  |  |  |  |
| E1 | 0.79 (0.22-2.90) | 0.723 | 0.86 (0.22-3.28) | 0.823 | 0.87 (0.23-3.33) | 0.835 | 0.87 (0.23-3.37) | 0.843 | 0.95 (0.24-3.68) | 0.936 |
| Segmental Glomerulosclerosis |  |  |  |  |  |  |  |  |  |  |
| S1 | 1.54 (0.46-5.13) | 0.483 | 1.37 (0.40-4.70) | 0.618 | 1.32 (0.38-4.58) | 0.663 | 1.25 (0.36-4.36) | 0.722 | 1.30 (0.38-4.47) | 0.677 |
| Tubular atrophy/interstitial fibrosis |  |  |  |  |  |  |  |  |  |  |
| T1 | 3.72 (1.01-13.54) | 0.048 | 3.69 (0.96-14.20) | 0.058 | 3.60 (0.91-14.28) | 0.068 | 3.56 (0.88-14.42) | 0.075 | 3.29 (0.79-13.71) | 0.103 |
| T2 | 1.62 (0.15-17.41) | 0.689 | 1.44 (0.13-16.65) | 0.770 | 1.34 (0.11-16.44) | 0.817 | 1.10 (0.09-14.20) | 0.943 | 0.996 (0.08-13.10) | 0.997 |
| At the time of biopsy |  |  |  |  |  |  |  |  |  |  |
| eGFR | 0.97 (0.95-0.997) | 0.028 | 0.97 (0.95-0.998) | 0.034 | 0.98 (0.95-1.001) | 0.058 | 0.98 (0.95-1.001) | 0.063 | 0.98 (0.95-1.003) | 0.078 |
| Gender (male) | 4.02 (0.97-16.72) | 0.056 | 5.18 (1.13-23.79) | 0.035 | 5.45 (1.13-26.19) | 0.034 | 6.53 (1.25-34.03) | 0.026 | 6.35 (1.22-33.11) | 0.028 |
| Age | 0.97 (0.92-1.03) | 0.323 | 0.97 (0.92-1.03) | 0.289 | 0.97 (0.92-1.03) | 0.338 | 0.97 (0.91-1.03) | 0.249 | 0.96 (0.90-1.02) | 0.170 |
| UA | 1.21 (0.89-1.63) | 0.224 | 1.23 (0.91-1.68) | 0.184 | 1.25 (0.91-1.72) | 0.171 | 1.26 (0.91-1.73) | 0.165 | 1.27 (0.91-1.76) | 0.158 |
| Hb | 0.87 (0.62-1.21) | 0.398 | 0.78 (0.54-1.13) | 0.192 | 0.77 (0.52-1.13) | 0.181 | 0.72 (0.48-1.10) | 0.127 | 0.71 (0.47-1.08) | 0.106 |
| Alb | 0.44 (0.20-0.96) | 0.040 | 0.61 (0.25-1.47) | 0.272 | 0.75 (0.29-1.91) | 0.542 | 0.72 (0.28-1.86) | 0.503 | 0.71 (0.28-1.82) | 0.474 |
| TC |  |  | 1.01 (0.998-1.02) | 0.101 | 1.01 (0.997-1.02) | 0.183 | 1.01 (0.997-1.02) | 0.138 | 1.01 (0.998-1.02) | 0.133 |
| 24h urine protein |  |  |  |  | 1.20 (0.93-1.53) | 0.156 | 1.16 (0.90-1.50) | 0.248 | 1.16 (0.90-1.49) | 0.255 |
| Hypertension (yes) |  |  |  |  |  |  | 1.77 (0.50-6.26) | 0.374 | 1.85 (0.51-6.67) | 0.347 |
| Macrohematuria (yes) |  |  |  |  |  |  |  |  | 0.14 (0.01-2.42) | 0.173 |
| Follow-up |  |  |  |  |  |  |  |  |  |  |
| TA-UA |  |  |  |  |  |  |  |  |  |  |
| TA-Hb |  |  |  |  |  |  |  |  |  |  |
| TA-Alb |  |  |  |  |  |  |  |  |  |  |
| TA-TC |  |  |  |  |  |  |  |  |  |  |
| TA-P |  |  |  |  |  |  |  |  |  |  |

continued

|  | Mode 11 |  | Mode 12 |  | Mode 13 |  | Mode 14 |  | Mode 15 |  |
| --- | --- | --- | --- | --- | --- | --- | --- | --- | --- | --- |
|  | OR(95%CI) | P | OR(95%CI) | P | OR(95%CI) | P | OR(95%CI) | P | OR(95%CI) | P |
| Mesangial hypercellularity |  |  |  |  |  |  |  |  |  |  |
| M1 | 6.31 (1.88-21.17) | 0.003 | 5.61 (1.47-21.49) | 0.012 | 5.10 (1.33-19.65) | 0.018 | 5.10 (1.33-19.60) | 0.018 | 7.79 (1.16-52.37) | 0.035 |
| Endocapillary hypercellularity |  |  |  |  |  |  |  |  |  |  |
| E1 | 1.14 (0.29-4.50) | 0.852 | 1.48 (0.32-6.82) | 0.612 | 1.62 (0.33-7.90) | 0.554 | 1.62 (0.33-8.02) | 0.557 | 0.62 (0.07-5.42) | 0.669 |
| Segmental Glomerulosclerosis |  |  |  |  |  |  |  |  |  |  |
| S1 | 0.95 (0.25-3.66) | 0.945 | 0.66 (0.14-3.01) | 0.587 | 0.56 (0.12-2.69) | 0.466 | 0.57 (0.12-2.74) | 0.480 | 0.58 (0.06-5.25) | 0.627 |
| Tubular atrophy/interstitial fibrosis |  |  |  |  |  |  |  |  |  |  |
| T1 | 1.97 (0.42-9.29) | 0.345 | 1.57 (0.29-8.47) | 0.599 | 1.72 (0.32-9.20) | 0.529 | 1.82 (0.33-10.0) | 0.492 | 2.89 (0.33-25.06) | 0.336 |
| T2 | 0.50 (0.04-6.93) | 0.605 | 0.22 (0.01-3.62) | 0.291 | 0.31 (0.02-5.27) | 0.418 | 0.32 (0.02-5.48) | 0.431 | 0.06 (0.001-3.75) | 0.184 |
| At the time of biopsy |  |  |  |  |  |  |  |  |  |  |
| eGFR | 0.97 (0.94-0.998) | 0.038 | 0.96 (0.93-0.996) | 0.028 | 0.96 (0.93-0.997) | 0.035 | 0.97 (0.93-0.998) | 0.039 | 0.97 (0.92-1.01) | 0.127 |
| Gender (male) | 4.16 (0.73-23.83) | 0.109 | 5.34 (0.82-34.92) | 0.080 | 5.28 (0.81-34.41) | 0.082 | 5.38 (0.82-35.49) | 0.081 | 7.36 (0.54-100.12) | 0.134 |
| Age | 0.93 (0.86-1.01) | 0.082 | 0.91 (0.83-1.01) | 0.067 | 0.92 (0.83-1.01) | 0.078 | 0.92 (0.83-1.01) | 0.087 | 0.84 (0.72-0.98) | 0.028 |
| UA | 0.87 (0.59-1.29) | 0.489 | 0.92 (0.60-1.41) | 0.696 | 0.92 (0.59-1.46) | 0.732 | 0.94 (0.59-1.50) | 0.795 | 1.15 (0.51-2.61) | 0.741 |
| Hb | 0.69 (0.44-1.09) | 0.111 | 1.17 (0.60-2.29) | 0.651 | 1.13 (0.59-2.19) | 0.708 | 1.14 (0.60-2.17) | 0.692 | 1.03 (0.40-2.65) | 0.956 |
| Alb | 0.75 (0.28-2.0) | 0.560 | 0.47 (0.16-1.39) | 0.172 | 0.52 (0.17-1.58) | 0.249 | 0.53 (0.18-1.62) | 0.267 | 0.47 (0.10-2.13) | 0.325 |
| TC | 1.01 (0.999-1.02) | 0.079 | 1.01 (0.995-1.02) | 0.276 | 1.01 (0.99-1.02) | 0.433 | 1.003 (0.99-1.02) | 0.687 | 1.02 (0.99-1.04) | 0.209 |
| 24h urine protein | 1.16 (0.91-1.49) | 0.233 | 1.18 (0.88-1.59) | 0.273 | 1.17 (0.87-1.58) | 0.292 | 1.17 (0.86-1.58) | 0.313 | 0.64 (0.33-1.24) | 0.183 |
| Hypertension (yes) | 2.58 (0.59-11.22) | 0.206 | 3.63 (0.71-18.53) | 0.121 | 4.20 (0.77-22.96) | 0.098 | 4.33 (0.79-23.80) | 0.092 | 16.38(0.93-288.49) | 0.056 |
| Macrohematuria (yes) | 0.11 (0.004-3.16) | 0.196 | 0.07 (0.001-6.48) | 0.246 | 0.05 (0-9.03) | 0.264 | 0.05 (0-9.02) | 0.264 | 0.01 (0-7.90) | 0.178 |
| Follow-up |  |  |  |  |  |  |  |  |  |  |
| TA-UA | 2.15 (1.25-3.70) | 0.006 | 2.14 (1.17-3.92) | 0.014 | 2.13 (1.14-3.97) | 0.017 | 2.06 (1.09-3.90) | 0.026 | 2.28 (0.72-7.28) | 0.164 |
| TA-Hb |  |  | 0.47 (0.28-0.81) | 0.006 | 0.53 (0.30-0.92) | 0.024 | 0.53 (0.30-0.91) | 0.022 | 0.47 (0.20-1.11) | 0.085 |
| TA-Alb |  |  |  |  | 0.45 (0.10-2.13) | 0.314 | 0.48 (0.10-2.37) | 0.367 | 1.34 (0.13-14.23) | 0.806 |
| TA-TC |  |  |  |  |  |  | 1.004 (0.99-1.02) | 0.697 | 0.999 (0.97-1.03) | 0.950 |
| TA-P |  |  |  |  |  |  |  |  | 18.68 (3.48-100.44) | 0.001 |

Stepwise regression: Variates were enrolled in the mode one by one or by category.
