# Supplementary material for: Risk factors of progressive IgA nephropathy which progress to end stage renal disease within ten years: a case–control study
Source: BMC Nephrol. 2017 Jan 7;18:11. doi: 10.1186/s12882-016-0429-x (PMC5219698; doi:10.1186/s12882-016-0429-x)
Supplement: Additional file 2: — Correlation between variates. Correlation analysis between each variate involved in this study. (DOCX 40 kb) [file 12882_2016_429_MOESM2_ESM.docx]

Additional file 2 Correlation between variates

|  |  |  |  |  |  |  |  |  |  |  |  |  |  |  |  |  |  |  |  |
| --- | --- | --- | --- | --- | --- | --- | --- | --- | --- | --- | --- | --- | --- | --- | --- | --- | --- | --- | --- |
|  | M | E | S | T | eGFR | Gender | Age | UA | Hb | Alb | TC | 24h urine protein | Hypertension | Macrohematuria | TA-UA | TA-Hb | TA-Alb | TA-TC | TA-P |
| M | 1 |  |  |  |  |  |  |  |  |  |  |  |  |  |  |  |  |  |  |
| E | -0.100 | 1 |  |  |  |  |  |  |  |  |  |  |  |  |  |  |  |  |  |
| S | 0.287** | 0.216** | 1 |  |  |  |  |  |  |  |  |  |  |  |  |  |  |  |  |
| T | 0.306** | 0.024 | 0.302** | 1 |  |  |  |  |  |  |  |  |  |  |  |  |  |  |  |
| eGFR | -0.323** | -0.184* | -0.193* | -0.629** | 1 |  |  |  |  |  |  |  |  |  |  |  |  |  |  |
| Gender | 0.120 | -0.269** | 0.032 | 0.00 | -0.003 | 1 |  |  |  |  |  |  |  |  |  |  |  |  |  |
| Age | -0.022 | -0.036 | -0.039 | 0.025 | -0.321** | -0.127 | 1 |  |  |  |  |  |  |  |  |  |  |  |  |
| UA | 0.388** | 0.043 | 0.196* | 0.442** | -0.482** | -0.369** | 0.054 | 1 |  |  |  |  |  |  |  |  |  |  |  |
| Hb | -0.064 | -0.273** | -0.102 | -0.263** | 0.247** | -0.460** | -0.144 | 0.037 | 1 |  |  |  |  |  |  |  |  |  |  |
| Alb | -0.198* | -0.223** | -0.317** | -0.319** | 0.250** | -0.274** | -0.137 | -0.110 | 0.587** | 1 |  |  |  |  |  |  |  |  |  |
| TC | 0.121 | 0.047 | 0.236** | 0.149 | -0.075 | 0.129 | 0.116 | 0.070 | -0.115 | -0.387** | 1 |  |  |  |  |  |  |  |  |
| 24h urine protein | 0.317** | 0.191* | 0.331** | 0.445** | -0.437** | 0.131 | 0.123 | 0.254** | -0.423** | -0.675** | 0.412** | 1 |  |  |  |  |  |  |  |
| Hypertension | 0.112 | -0.147 | 0.035 | 0.223** | -0.212** | -0.019 | 0.282** | 0.144 | 0.139 | -0.012 | 0.067 | 0.147 | 1 |  |  |  |  |  |  |
| Macrohematuria | -0.116 | 0.048 | -0.058 | -0.137 | 0.220** | 0.086 | -0.281** | -0.160 | -0.027 | 0.031 | -0.128 | -0.172* | -0.173* | 1 |  |  |  |  |  |
| TA-UA | 0.365** | -0.053 | 0.220** | 0.452** | -0.402** | -0.372** | -0.037 | 0.767** | 0.008 | -0.114 | 0.048 | 0.263** | 0.132 | -0.205* | 1 |  |  |  |  |
| TA-Hb | -0.188* | -0.125 | -0.148 | -0.360** | 0.302** | -0.362** | -0.148 | -0.052 | 0.726** | 0.427** | -0.178* | -0.417** | 0.025 | 0.104 | -0.106 | 1 |  |  |  |
| TA-Alb | -0.281** | 0.004 | -0.181* | -0.226** | 0.173* | -0.103 | -0.053 | -0.087 | 0.402** | 0.560** | -0.300** | -0.437** | 0.027 | 0.035 | -0.189* | 0.536** | 1 |  |  |
| TA-TC | 0.166* | 0.049 | 0.149 | 0.079 | -0.011 | -0.089 | 0.021 | -0.024 | -0.134 | -0.371** | 0.671** | 0.413** | -0.018 | -0.050 | 0.042 | -0.183* | -0.387** | 1 |  |
| TA-P | 0.341** | 0.144 | 0.267** | 0.467** | -0.462** | -0.009 | 0.055 | 0.379** | -0.294** | -0.445** | 0.225** | 0.596** | 0.130 | -0.164* | 0.432** | -0.394** | -0.417** | 0.281** | 1 |

Correlation between variates were analyzed using spearmen correlation analysis. *P<0.05, **P<0.01.
